# Care homes’ use of medicines study: prevalence, causes and potential harm of medication errors in care homes for older people

**DOI:** 10.1136/qshc.2009.034231

**Published:** 2009-09-25

**Authors:** N D Barber, D P Alldred, D K Raynor, R Dickinson, S Garfield, B Jesson, R Lim, I Savage, C Standage, P Buckle, J Carpenter, B Franklin, M Woloshynowych, A G Zermansky

**Affiliations:** 1 Department of Practice and Policy, School of Pharmacy, London, UK; 2 School of Healthcare, University of Leeds, Leeds, UK; 3 Robens Centre for Public Health, University of Surrey, Guildford, Surrey, UK; 4 London School of Hygiene and Tropical Medicine, London, UK; 5 Imperial College Healthcare NHS Trust, London, UK

## Abstract

**Introduction:**

Care home residents are at particular risk from medication errors, and our objective was to determine the prevalence and potential harm of prescribing, monitoring, dispensing and administration errors in UK care homes, and to identify their causes.

**Methods:**

A prospective study of a random sample of residents within a purposive sample of homes in three areas. Errors were identified by patient interview, note review, observation of practice and examination of dispensed items. Causes were understood by observation and from theoretically framed interviews with home staff, doctors and pharmacists. Potential harm from errors was assessed by expert judgement.

**Results:**

The 256 residents recruited in 55 homes were taking a mean of 8.0 medicines. One hundred and seventy-eight (69.5%) of residents had one or more errors. The mean number per resident was 1.9 errors. The mean potential harm from prescribing, monitoring, administration and dispensing errors was 2.6, 3.7, 2.1 and 2.0 (0 = no harm, 10 = death), respectively. Contributing factors from the 89 interviews included doctors who were not accessible, did not know the residents and lacked information in homes when prescribing; home staff’s high workload, lack of medicines training and drug round interruptions; lack of team work among home, practice and pharmacy; inefficient ordering systems; inaccurate medicine records and prevalence of verbal communication; and difficult to fill (and check) medication administration systems.

**Conclusions:**

That two thirds of residents were exposed to one or more medication errors is of concern. The will to improve exists, but there is a lack of overall responsibility. Action is required from all concerned.

## Introduction

Older people living in care homes are potentially at greater risk of medication error than most other groups. They are prescribed multiple medicines and this, coupled with age-related changes in pharmacokinetics and pharmacodynamics, makes them particularly susceptible to adverse drug events.^1^ ^2^ Many have some degree of cognitive impairment that prevents them from being actors in the detection of errors.^3^ In addition, the medicines management system in care homes (described in box 1) is complex—for example, many residents receive clinical interventions from multiple sources; medicines may be dispensed from multiple sites; and medicines may be dispensed in, and administered from, one of several different types of packaging systems known as “monitored dosage systems” (MDS).

In 2000, a report documenting the extent of medical error was published and the UK government committed to reducing errors^4^ ^5^; medication errors were a particular concern. Prescribing has been found to be suboptimal in UK care homes^6^ ^7^; however, we do not know the prevalence of medication errors in this setting in the UK. Such studies in the USA have been undertaken; however, they have often relied on spontaneous reporting,^8^ ^9^ which significantly underestimates the prevalence of error.^10^ ^11^ A US study of medication administration errors using direct observation found an error rate of 22% in nursing homes.^12^ Gurwitz *et al*^2^ found 4.1 preventable adverse drug events per 100 resident-months in two US long-term care facilities.

Although previous studies have measured prevalence of specific types of medication errors, they have not been designed to look at all types of error and simultaneously understand their causes. The theory of causation of human error^13^ ^14^ ^15^ is widely used in healthcare and has been used to explain causes of medication errors previously.^16^ ^17^ In this study, we wished to determine the prevalence of all forms of medication errors in care homes, to assess the potential of these errors for harm and to establish the underlying causes.

## Methods

Ethical approval was obtained from the Central Office for Research Ethics Committees.

### Selection criteria/participants

We chose to sample a large number of homes and small number of residents per home. It is generally acknowledged that errors are a consequence of the systems being used,^15^ so our sampling strategy was designed to increase the number of systems of care observed (homes, general practitioners (GPs) and pharmacies). Care homes for older people (nursing, residential and mixed) were sampled in three geographically spread and demographically diverse areas in the UK (West Yorkshire, Cambridgeshire and central London) to obtain a varied sample with respect to ownership, size and type of care provided. For each home we approached the manager/head office to obtain cooperation and obtained written consent from care home staff who were observed. Care home residents on one or more medicines were randomly selected and included in the study if they provided written, informed consent. For those lacking capacity, assent was obtained from the next of kin. Written, informed consent was obtained from GPs providing a service to the homes, which included access to their care home records and GP records.

### Data collection

We used a mixture of methods. The qualitative work was ethnographically informed and involved field notes, observation and semistructured interviews based on Reason and Vincent’s frameworks.^14^ ^15^ To help understand the causes of specific errors, we conducted interviews with the home staff, GP or pharmacist. Interviews were taped where possible and transcribed; otherwise, notes were made that were expanded on after the interview. Analysis of the causes of errors used Reason’s framework.^14^ ^18^

The quantitative work to identify prescribing and monitoring errors was undertaken by clinical pharmacists (one in each of the three areas) conducting clinical medication reviews for each randomly selected resident. This process included review of GP and care home notes and consultation with the resident and/or staff.^1^ Dispensing errors were identified by comparing the prescriptions and medication administration record sheets with the dispensed medicines. For each drug, it was noted if it was contained in an MDS or not, and if so, whether it was in a cassette MDS or blister-pack MDS. Two drug rounds per resident were observed to identify administration errors. The definitions and denominators of the four types of error are described in appendix A. To ensure inter- and intrarater reliability, we produced a handbook of definitions and procedures, and research staff were trained in homes together and met regularly to resolve difficult cases, which were then added to the handbook. If any errors were thought to be likely to cause patient harm, then the pharmacist intervened.

The potential harm of each error was assessed using a valid and reliable method.^19^ Each error was individually assessed by a GP, a consultant old age psychiatrist, a clinical pharmacologist and two clinical pharmacists, using a validated 10-point scale (0 = no harm; 10 = death); their mean score was taken as the harm score for that error.

### Statistical analysis

Sample size was calculated to give a precision of +/−2% SD (95% confidence interval (CI)) if the prevalence of prescribing error was 10%. The estimation was derived from a combination of simulation and formulas for clustered data, and the output was a matrix of numbers of homes and number of patients per home (eg, 100 homes with three patients each gave a precision of 1.9%; 50 homes with six patients each gave a precision of 2.04%), allowing us to adapt the sample dependent on recruitment rates. Statistical analysis was performed using the software R 2.3 (R project for statistical computing, www.r-project.org). Statistical “significance” was predefined at the 5% level. Exact binomial CIs were calculated for proportions. χ^2^ tests were used to assess differences in error rates between areas. Generalised estimating equations (library geepack, V.1.0-10) were used to model patient level odds of errors, allowing for clustering in homes and using an independence correlation structure. Multilevel models were also used to model patient level odds of errors, using the MLwiN 2.03 software (multilevel models project, University of Bristol, http://www.cmm.bristol.ac.uk), fitting variance components at the various levels.

## Results

Of those approached, 72% (79/108) of homes, 67% (269/399) of residents and 61% (54/89) of general practices agreed to take part. Two hundred and fifty-six residents were recruited from 55 care homes. The majority (38, 69%) of the homes provided both residential and nursing care (corresponding figures for residential care only and nursing care only: 12 (22%) and 5 (9%)). Table 1 shows demographic data. The majority of the residents were women (69%, 177/256) and very old (mean age 85 years). There were slightly more residential care residents (54%, 139/256) than nursing care residents. Two hundred and twenty (86%) of the residents were dispensed some of their medicines in a monitored dosage system. There was a mean of 3.8 (range 1–14) GP practices per care home. There was considerable variation between areas: in London, the median was 1 (range 1–3) practice per home; in West Yorkshire, the median was 5 (range 1–14).

**Table 1:** Demographic data

No of residents approached	399
No of residents consented/assented (%)	269 (67.4)
No of residents excluded	13
Consent given post cut-off	6
Died	4
In hospital	3
No of residents entered into study	256
Cambridgeshire	31
West Yorkshire	121
London	104
Women, no (%)	177 (69.1)
Age (y), mean (range)	85.2 (60–102)
Nursing residents, no (%)	117 (45.7)
Residential residents, no (%)	139 (54.3)
Mean no of medicines per resident (95% CI)	8.0 (7.5 to 8.5)
Median no of medicines per resident (range)	7.5 (1–25)
No of residents using monitored dosing systems (%)	220 (85.9)
Cambridgeshire (%)	23 (74.2)
West Yorkshire (%)	117 (96.7)
London (%)	80 (76.9)

We intended to interview those involved in specific errors, soon after they had happened. However, many errors (such as some prescribing errors) had been made some time in the past, so most interviews with doctors and pharmacists focused more on problems in general in medicines prescribing, dispensing and use in care homes. We undertook 59 interviews relating to 66 administration errors, 34 dispensing errors, 18 prescribing and 8 monitoring errors. Further general interviews were carried out with 19 pharmacists and 11 GPs, and observations were carried out in five pharmacies.

Table 2 details the prevalence and potential harm of errors. Overall, 178 of 256 residents (69.5%, 95% CI 63.5 to 75.1) had at least one medication error (prescribing, monitoring, administration or dispensing). There was a mean of 1.9 (95% CI 1.64 to 2.17) errors per resident. Appendix B gives examples of errors.

**Table 2:** Error prevalence and harm assessment

	Type of error				Overall
	Prescribing	Monitoring	MAE	Dispensing	
Number of residents with an error (n = 256) (%; 95% CI)	100 (39.1%; 33.0 to 45.3)	27 (10.5%; 7.1 to 15.0)	57 (22.3%; 17.3 to 27.9)	94 (36.7%; 30.8 to 42.9)	178 (69.5%; 63.5 to 75.1)
Mean number of errors per resident (95% CI)	0.60 (0.48 to 0.71)	0.13 (0.08 to 0.17)	0.45 (0.32 to 0.58)	0.73 (0.56 to 0.90)	1.91 (1.64 to 2.17)
Median number of errors per resident (range)	0 (0–6)	0 (0–3)	0 (0–7)	0 (0–9)	1 (0–13)
Number of errors/opportunity for error (%; 95% CI)	153/1837 (8.3%; 7.1 to 9.7)	32/218 (14.7%; 10.3 to 20.1)	116/1380 (8.4%; 7.0 to 10.0)	187/1915 (9.8%; 8.5 to 11.2)	488/5350 (9.1%; 8.4 to 9.9)
Mean harm score* (95% CI)	2.6 (2.4 to 2.8)	3.7 (3.4 to 4.0)	2.1 (1.9 to 2.3)	2.0 (1.8 to 2.2)	2.6 (2.5 to 2.7)
Median harm score* (range)	2 (0.2 to 5.8)	3 (2.8 to 5.2)	2 (0.1 to 5.8)	1 (0.2 to 6.6)	2 (0.1 to 6.6)

MAE, medication administration error.

*Assessed by a 10-point scale^19^ (0 = no harm; 10 = death).

### Prescribing and monitoring errors

One hundred residents (39.1%, 95% CI 33.0 to 45.3) had one or more prescribing errors, totalling 153 prescribing errors, and the prescribing error rate by opportunity for error was 8.3% (95% CI 7.1 to 9.7) (opportunity for error is an act that can be erroneous; in this case it would be the prescribing of one medicine or the monitoring of one). The most common types of prescribing error, accounting for 87.6% of the total, were categorised as “incomplete information” (37.9% meaning, eg, that no strength or route was specified when there was more than one option), “unnecessary drug” (23.5%), “dose/strength error” (14.4%) and “omission” (11.8%).

In total, there were 147 residents who were prescribed a medicine that required monitoring, and 18.4% (27) of these had an error. There were 32 monitoring errors in the 218 prescribed items that required monitoring (14.7%). The mean number of monitoring errors per resident was 0.13 (95% CI 0.08 to 0.17). There was significant variation between areas, with 75% of monitoring errors occurring in just one geographical area (p<0.01). Nearly one third (30.8%) of medicines deemed to require monitoring in the problem area were not being monitored. The great majority of monitoring errors (90.6%) resulted from a failure to request monitoring. The drugs most commonly associated with monitoring errors were diuretics (53.1%), ACE inhibitors (15.6%), amiodarone (12.5%) and levothyroxine (9.4%). The mean harm scores for prescribing and monitoring errors were 2.6 (range 0.2 to 5.8) and 3.7 (range 2.8 to 5.2), respectively.

The interviews categorised factors thought to contribute to the errors as the patient, the task, the team and the work environment. Patient factors related to prescribing errors included that patients were generally home bound and could not access services external to the home; some patients also disliked blood tests or taking some medicines. Some patients were confused and unable to give histories. Task factors related to lack of usual prescribing technical support, including computer-based aids and accessing the medical record. There were problems with practice computers’ failure to prompt monitoring tests clearly. It could be hard to get blood tests done. There were many “team factors” as there was little sense of being a whole team (home, pharmacy and practice staff). The service offered by GP practices was very variable, from a dedicated GP making weekly visits, to GPs with no knowledge of the patient making a home visit when requested. Hospital out-patient and discharge letters were sometimes unclear, delayed, missed or not adequately incorporated in the patient record. When changing between GP practices, patient notes took up to 4 months to arrive. GPs often expressed concern about the care home staff, including turnover and staff shortages. The skills of care home staff were sometimes seen as low, which several linked to pay.

### Medication administration errors

Fifty-seven (22.3%, 95% CI 17.3 to 27.9) residents had a total of 116 administration errors. The mean number of administration errors per resident was 0.45 (95% CI 0.32 to 0.58) and the prevalence of administration errors by opportunities for error was 8.4% (95% CI 7.0 to 10.0). Nearly half (49.1%) of all administration errors were categorised as “omissions” and just more than one fifth (21.6%) were “wrong dose”. The odds of a medication administration error occurring were higher in residential care than in nursing care residents; however, this just failed to reach statistical significance at the 5% level (adjusted OR 1.77, 95% CI 0.96 to 3.25, p = 0.063; adjusted for age, sex and medication delivery system). The higher apparent risk of administration errors in residential compared with nursing residents was largely attributable to more “omissions” (38 vs 19) and “wrong doses” (18 vs 7). There was no statistically significant difference in the administration errors by the medicine delivery system. The mean harm score was 2.1 (range 0.1–5.8).

Patient factors included many patients’ lack of awareness of their medicines. In addition, their physical condition could make it hard to administer medicines properly. Some patients had fears about medicines, such as feeling they were being poisoned, and some were consequently aggressive. Finding mobile patients during the drug round could be a problem. Changing or adding medicines, such as acute treatment, in the middle of the 4-week supply cycle could be a problem. Task factors included inability to find the medicine, failure to order the right quantity of “as required medicines”, the special requirements that some medicines had (eg, “take on an empty stomach”), the difficulty many staff had in correctly administering inhalers and a lack of adequate protocols. Individual factors related to the staff included lack of knowledge about inhalers and the timing of medicines with respect to food. Team factors included the medication administration record chart, which should be the documentary line of communication among GP, home and pharmacist; these records were often inaccurate. Communication within the home tended to be verbal. Work environment factors included homes being hot, airless, having unpleasant smells, being poorly lit, noisy and short of space. There were often staffing problems in the morning round (when most medicines were given and when staff also had most other tasks). Staff were frequently interrupted and did not have dedicated time to order medicines. In some homes, 12-hour shifts were usual.

### Dispensing errors

Ninety-four residents (36.7%, 95% CI 30.8 to 42.9) had a total of 187 dispensing errors with a mean of 0.73 (95% CI 0.56 to 0.90) dispensing errors per resident. The dispensing error rate by opportunity for error was 9.8% (95% CI 8.5 to 11.2). Labelling errors were found in 7.3% of dispensed items, content errors in 2.3% and clinical errors in 0.21%. There was a borderline statistically significant difference in the odds of a dispensing error according to delivery system (p = 0.056), largely due to the higher odds with the cassette type monitored dosage systems (cassette vs blister: adjusted odds ratio, 2.88, 95% CI 1.5 to 5.55, p = 0.0012), which was associated with more labelling errors. The mean harm score was 2.0 (range 0.2–6.6).

Task factors included the computer systems used, with some identifying all interactions rather than the clinically significant ones. Some monitored dosage systems did not have the space to fit in all the required warning labels, and they were tedious and difficult to fill. The similar appearance of many tablets when removed from their original container was a potential source of error. The prescription and medication administration record were often different, so it was unclear which was correct. Individual factors included staff feeling hungry, tired or unwell while dispensing monitored dosage systems. There was lack of knowledge by pharmacy staff of the care homes’ systems and their need for support. Team factors overlapped with this—some pharmacists had no knowledge of the care home and its requirements, relationships were sometimes tense and poor language skills of home staff were sometimes cited. Some pharmacies had a poor checking process, and use of locums could be a problem. Work factors included the pharmacies being seen as being busy and pressured, with interruptions and distractions (including noise) and some staff shortages.

### Organisational culture

When considering all forms of medication errors, factors relating to organisational culture (called “latent failures” by Reason^20^) were significant. It was clear from the interviews that no one took responsibility for the whole system. We often saw well-intentioned people doing their best but in an uncoordinated way. Communication, written and verbal, was another problematic factor, within and among the home, GP practice and pharmacy. Consequently, it is difficult to know which medicines any patient should be having. Management within each organisation was a factor, particularly when challenged to deliver a safe service within a tight budget.

## Discussion

People in care homes are a frail and vulnerable population at particular risk from medication errors, and it is a cause for concern that two thirds of care home residents in this study were exposed to one or more errors. For each event involving prescribing, dispensing or administration of a medicine, there was an 8%–10% chance of an error happening and a 14% chance of a monitoring error. Safety is a systems issue, and we believe this is the first study to consider the whole system of medication use in care homes; our simultaneous collection of qualitative data has allowed us to understand the causes of error and suggest solutions.

The prevalence of prescribing error is similar to that found in primary care^21^; administration error prevalence was a little higher than that in hospital^22^ (and likely to be better than the patients’ adherence if in their own home).^23^ The prevalence of dispensing errors was three times higher than the rate found in primary care in the UK,^24^ although that study excluded MDS. Our higher rate predominantly reflected one type of MDS that was difficult to label fully.

Although our study was not primarily designed to identify the prevalence of harm, we saw several errors, particularly monitoring errors, which had caused harm or were likely to. In addition, many errors would reduce the quality of life and ability to function of residents, such as inadequate treatment of pain, of bowels and of breathing.

Limitations to our study include that our sample only contained those willing to be studied (although the acceptance rate of homes was high) and that our home sampling was not random. Judgement of the cause of error was sometimes difficult as there could be conflicting sources of evidence or a lack of evidence; hence, judgements sometimes retained an element of subjectivity. Observation may theoretically have affected the prevalence of administration error, although routine observation has been found to have no effect.^11^ Staff interviewed will have given accounts affected by hindsight bias; hence, imputations of causality are speculative.

What can be done, and who should do it? As our study shows, there are currently many and varied subsystems that are not being seen in an integrated way. There is now the opportunity for a systems approach to the whole. Since 2008, chief pharmacists of provider organisations and commissioners in England should have the lead role in ensuring safe medication practices are embedded in patient care^25^; a significant and pressing agenda for them. An additional system-based solution relates to several of the communication and records problems observed—we would hope these would be ameliorated by programmes in the National Health Service’s information technology programme (NPfIT), such as the Summary Care Record (a brief GP record which can be accessed by others), GP2GP (electronic transfer of patients’ notes between GPs) and the Electronic Prescription Service (electronic transfer of primary care prescriptions). The final system issue is that most primary care is based on patients going to centres of care rather than the other way around. Primary care services that are based on care going to patients need to be commissioned, in order not to disadvantage the home bound.

We suggest the idea of a lead (not sole) GP for each home should be explored. This role would need protected time and associated funding. In addition to caring for patients, they should liaise with other GPs and have responsibility to ensure, possibly by commissioning services, that patients on riskier medicines are appropriately monitored and that all patients’ medication is regularly reviewed by a pharmacist.

Consideration should be given to having one person with overall responsibility for medicines use in one or more care homes. Many pharmacists have the skills and knowledge to undertake this role, and such developments are described in the UK government’s recent proposals for making best use of pharmacists’ expertise.^25^

Pharmacists supplying homes should ideally know the home, its ways and needs, so that ordering and supply match the home’s (and patients’) needs. The widespread use of MDS unit dose systems, requiring millions of tablets to be repackaged each week, is a vast, unfunded undertaking. It imposes demands on home and pharmacy alike, yet its contribution to safety is unclear and some commissioners discourage its use. The use of MDS drives efficiencies of scale, such as large centralised repackaging units, which in turn leads to the dispensary becoming remote from the customers (home and patient). Research into the effectiveness of MDS is urgently required.

Within homes the use and accuracy of the medication administration record requires constant review. The lack of protocols and adequate staff training remains an issue. Drug rounds are very busy, and often interrupted in the morning, and some medicines should be prescribed for different times to ease this. The commonest administration errors were omissions because the drug was not available, so omissions need to be monitored and ordering, particularly of “as required” medicines, needs to be improved.

We were very impressed by the proportion of homes participating in a study, which was potentially very threatening to them. Several care home managers have told us that patient harm from medication error is their greatest fear and that up to half of staff time can be spent on medication related activities. Given this motivation and resource, we are hopeful of change.

**Box 1**

**Context: Care homes and the English National Health Service**

Care homes may provide 24-hour nursing care (nursing homes), personal care only (residential homes) or a combination. They may be owned by individuals or companies of various sizes, including large private health providers, charities or by the local authorities. Care homes are reviewed against standards by the Care Quality Commission, which is the independent regulator of health and social care in England. Each resident is registered with a general practitioner who provides their medical care and keeps their medical record. When a patient transfers from their own home to a care home, they can elect to keep their general practitioner if he or she is local.

In England, the National Health Service is delivered through 152 primary care trusts, which are responsible for a geographical area of the country, for which they commission healthcare from general practitioners (primary care physicians) who usually work as part of a group practice, pharmacies and others. Pharmacies may be owned and run by a single pharmacist or may be part of a chain; the largest chains are run by international companies. Homes usually obtain their regular supply of medicines for all their residents from one pharmacy.

Repeat medicines are ordered from the GP practice (usually monthly) by the care home staff using the previous 28-day medicine administration record or the repeat medicines slip provided by the GP practice. Generally the GP practice authorises and prints the repeat prescriptions and sends them to the care home for checking. They are then forwarded to the community pharmacy where they are dispensed and delivered to the care home with a new 28-day medicine administration record.

## Appendix A: Error definitions

*Prescribing errors* were identified and classified according to the definition developed by Dean *et al*^26^ as “A prescribing decision or prescription-writing process that results in an unintentional, significant: reduction in the probability of treatment being timely and effective; or increase in the risk of harm, when compared with generally accepted practice”. The number of opportunities for error (denominator) was the number of prescription items written, plus any omissions. The three pharmacists worked to a common detailed protocol when reviewing the residents and their medicines, were trained together at the start of the study and had regular review meetings to ensure consistency.

*Monitoring errors* were identified according to the definition developed and validated by Alldred *et al* 27 as “A monitoring error occurs when a prescribed medicine is not monitored in the way which would be considered acceptable in routine general practice. It includes the absence of tests being carried out at the frequency listed, with a tolerance of +50%. This means—for example, that if a medicine requires liver function tests at three monthly intervals, an error would occur if a test was not conducted within 18 weeks”. The number of opportunities for error (denominator) was the number of prescribed items that required monitoring, according to the validated criteria.

The definition of a *dispensing error* developed by Beso *et al*^28^ was adopted. A dispensing error was defined as “One or more deviations from an interpretable written prescription or medication order, including written modifications to the prescription made by a pharmacist following contact with the prescriber”. Dispensing errors were identified and classified by the clinical pharmacists by comparing the prescriptions and medicine administration record sheets with the dispensed medicines. The number of opportunities for error (denominator) was the number of prescription items dispensed or omitted.

The clinical pharmacists observed two drug rounds per resident to identify and classify *medication administration errors* as defined using previous work by Allan and Barker^29^ and Dean and Barber^11^ as “any deviation between the medication prescribed and that administered”. The number of opportunities for error (denominator) was the number of doses given, plus any doses that should have been given but were omitted.

## Appendix B: Examples of errors

### Case 1: Prescribing error

Tramadol capsules 50 mg prescribed “one to be taken **up to** four times a day” for chronic foot pain, resident also taking warfarin for long-term DVT prophylaxis (Tramadol can enhance the effect of warfarin). International normalised ratio checked regularly with erratic results ranging from 0.9 to 4.5 (mean harm score 5.8).

### Case 2: Prescribing error

Donepezil 10-mg tablets prescribed “one daily” for dementia. Following a review from hospital specialist, a letter to the GP indicated Aricept (brand name for donepezil) should be stopped. The donepezil had not been discontinued by the GP and there was no indication in the medical notes that the continuation was deliberate (mean harm score 1.8).

### Case 3: Monitoring error

Lisinopril 5-mg tablets prescribed “one daily” for hypertension for a resident with an estimated creatinine clearance of 19 ml/min. A potassium level had been checked 1 year ago and had revealed a high potassium level 5.8 mmol/l (range 3.5–5 mmol/l): no action had been taken (mean harm score 5.8).

### Case 4: Monitoring error

Amiodarone 200-mg tablets prescribed “one daily” for atrial fibrillation. Thyroid function tests were last checked 9 months ago, when thyroid stimulating hormone was 12.9 mlU/l (range 0.3–5.5 mlU/l) and thyroxine was 18 nmol/l (range 50–151 nmol/l), and no action had been taken (mean harm score 5.8).

### Case 5: Medication administration error

Bendroflumethiazide 2.5-mg tablets prescribed “one each morning” for hypertension. This was discontinued due to low serum sodium of 127 mmol/l (range 135–145 mmol/l); however, it remained on the current medication administration record when the next monthly drug supply was made and hence continued to be administered (mean harm score 4.6).

### Case 6: Dispensing error

Aspirin enteric-coated 75 mg tablets dispensed instead of zopiclone 7.5 mg tablets as a 7-day supply in a cassette dispensing system (mean harm score 5.0).
